# Hierarchical Clustering Analyses of Plasma Proteins in Subjects With Cardiovascular Risk Factors Identify Informative Subsets Based on Differential Levels of Angiogenic and Inflammatory Biomarkers

**DOI:** 10.3389/fnins.2020.00084

**Published:** 2020-02-06

**Authors:** Zachary Winder, Tiffany L. Sudduth, David Fardo, Qiang Cheng, Larry B. Goldstein, Peter T. Nelson, Frederick A. Schmitt, Gregory A. Jicha, Donna M. Wilcock

**Affiliations:** ^1^Sanders-Brown Center on Aging, University of Kentucky, Lexington, KY, United States; ^2^Department of Physiology, University of Kentucky, Lexington, KY, United States; ^3^Department of Biostatistics, University of Kentucky, Lexington, KY, United States; ^4^Department of Computer Science, University of Kentucky, Lexington, KY, United States; ^5^Department of Neurology, University of Kentucky, Lexington, KY, United States; ^6^Department of Pathology, University of Kentucky, Lexington, KY, United States

**Keywords:** hierarchical clustering analysis, vascular cognitive impairment and dementia, mild cognitive impairment, VEGF, MMP1, IL8

## Abstract

Agglomerative hierarchical clustering analysis (HCA) is a commonly used unsupervised machine learning approach for identifying informative natural clusters of observations. HCA is performed by calculating a pairwise dissimilarity matrix and then clustering similar observations until all observations are grouped within a cluster. Verifying the empirical clusters produced by HCA is complex and not well studied in biomedical applications. Here, we demonstrate the comparability of a novel HCA technique with one that was used in previous biomedical applications while applying both techniques to plasma angiogenic (FGF, FLT, PIGF, Tie-2, VEGF, VEGF-D) and inflammatory (MMP1, MMP3, MMP9, IL8, TNFα) protein data to identify informative subsets of individuals. Study subjects were diagnosed with mild cognitive impairment due to cerebrovascular disease (MCI-CVD). Through comparison of the two HCA techniques, we were able to identify subsets of individuals, based on differences in VEGF (*p* < 0.001), MMP1 (*p* < 0.001), and IL8 (*p* < 0.001) levels. These profiles provide novel insights into angiogenic and inflammatory pathologies that may contribute to VCID.

## Introduction

Vascular cognitive impairment and dementia (VCID) is an active area in dementia research (Murphy et al., 2016) and is described as “encompassing all the cognitive disorders associated with cerebrovascular disease (CVD), from dementia to mild cognitive deficits” (Gorelick et al., 2011). VCID is estimated to occur in roughly 20% of the cases of dementia; however, the exact prevalence in the population is unknown with varying estimates in the literature (Rizzi et al., 2014; Corriveau et al., 2016). Much of the uncertainty in assessing the prevalence of VCID is due to varied diagnostic criteria (Harrison et al., 2016). In addition, there is substantial overlap in cognitive manifestations of cerebrovascular and neurodegenerative pathologies [such as Alzheimer’s disease (AD)] that can culminate in clinical dementia (Kapasi and Schneider, 2016), which further complicates our understanding of VCID. Further, both pathologies commonly co-exist in the same individual, yet some autopsy studies suggest that there is a significant increase in dementia risk due to vascular factors when Alzheimer pathology is low (Abner et al., 2017; Dodge et al., 2017).

Currently, magnetic resonance imaging (MRI) and cerebrospinal fluid (CSF) biomarkers are used to differentiate VCID from AD and monitor the progression of VCID (Cipollini et al., 2019; Wardlaw et al., 2019). Plasma biomarkers are currently being investigated as a lower cost and less invasive alternative approach. The current study is focused on exploring the potential clustering of plasma biomarkers using hierarchical clustering analysis (HCA) in participants with VCID who have mild cognitive impairment (MCI) due to CVD (MCI-CVD) to identify unique plasma profiles of disease (Winblad et al., 2004). Persons with MCI-CVD are of particular interest as they are at an increased risk of developing dementia and already have cognitive decline (Petersen et al., 1999). We evaluated angiogenic (FGF, FLT, PIGF, Tie-2, VEGF, VEGF-D) and inflammatory (MMP1, MMP3, MMP9, IL8, TNFα) protein plasma biomarkers in these participants using the highly sensitive meso-scale discoveries (MSD) platform. Angiogenic and inflammatory markers are of particular interest due to their roles in endothelial dysfunction, which has been shown to play a role in the pathogenesis of CVD (Poggesi et al., 2016; Mahoney et al., 2019). Presently, studies have demonstrated mixed results in the association of angiogenic and inflammatory biomarkers with VCID; however, it is suspected that this is due to the inconsistency in both the patient populations and the analytical measures (Cipollini et al., 2019).

Agglomerative HCA is an unsupervised machine learning technique commonly used to determine similar subsets within a larger population (Xu and Wunsch, 2010). HCA can be used to identify subsets within a variety of different patient populations. The accuracy of this technique is difficult to quantify, as most studies rely on *post hoc* analysis of the clusters produced by HCA to determine their validity. We propose a unique methodology for validating clusters produced by HCA. This method relies on using two unique HCA models on the same dataset and evaluates congruencies between the two models by comparing a novel HCA model to one that is widely used (Wallin et al., 2010; Damian et al., 2013; Nettiksimmons et al., 2014; Racine et al., 2016). Before applying both HCA models to our dataset, we tested the accuracy of each model on various distributions of data and compared them to each other using the adjusted rand index (ARI). After demonstrating the interchangeability of the two HCA models in the simulated data distribution comparable to our dataset, we tested both models on our dataset and compared the underlying components of each cluster produced by the HCA models.

## Materials and Methods

### Participants

Plasma samples were collected from a cohort of adult research volunteers enrolled in a randomized behavioral intervention study for MCI-CVD (*N* = 80, NCT01924312). Inclusion criteria for the parent study include age older than 55 years, Montreal Cognitive Assessment score < 29, and at least one uncontrolled vascular risk factor. Risk factors included poorly controlled hypertension, poorly controlled cholesterol, cardiomyopathy/CHF, diabetes with a fasting glucose > 110 or HbA1c > 7%, homocysteine > 12, history of transient ischemic attack, tobacco use > 30 pack-years, and BMI > 30. Potential subjects were excluded from this cohort if they had dementia, evidence of a non-CVD cause of cognitive decline, evidence of a non-CVD neurologic disease, or any focal motor, sensory, visual, or auditory deficits. For the current study, participants were also excluded if they had an incomplete panel of markers as measured via MSD assays as described below (*n* = 7).

### Plasma Collection and Quantification

Plasma samples were collected by venous puncture using 10 mL EDTA Vacutainer tubes. Plasma was aliquot into cryo-tubes at 500 mL volumes. Quantification of plasma samples was accomplished using MSD Multi-Spot V-PLEX assays [Angiogenesis Panel 1 (human) and Proinflammatory Panel 1 (human)] and Ultra-Sensitive assays (MMP 2-Plex and MMP 3-Plex). Plasma did not undergo any freeze-thaw cycles after the initial thawing of the aliquot. Assays were performed using plate specific protocols as followed with analysis performed in the MSD Discovery Workbench 4.0 software.

#### MMP 2-Plex and MMP 3-Plex

MMP plates were brought to room temperature for approximately 30 min and then loaded with 25 μL of diluent, covered (protect from light), and incubated at room temperature for 30 min while shaking at 600 r/min. After incubation, plates were removed from the shaker and 25 μL of calibrator was added to the assigned wells in duplicate with 5 μL of undiluted sample and 20 μL of diluent. Plates were covered and incubated at room temperature while shaking at 600 r/min. After incubation, plates were removed from the shaker and washed three times with 300 μL of wash buffer. Plates were turned upside down and tapped against paper towels to ensure the removal of all wash buffer from the wells. 25 μL of the antibody mix was loaded into each well, covered (protect from light), and incubated at room temperature for 2 h shaking at 600 r/min. After incubation, plates were removed from the shaker and the wash steps were repeated from above; 150 μL of read buffer was loaded into each well and read on the MSD Quickplex SQ 120 machine.

#### Proinflammatory Panel 1

Proinflammatory plates were brought to room temperature for approximately 30 min and washed three times with 300 μL of wash buffer. Plates were turned upside down and tapped against paper towels to ensure the removal of all wash buffer from the wells; 50 μL of calibrator was added to the assigned wells in duplicate with 50 μL of undiluted sample and covered (protect from light). Plates were incubated at 4°C overnight while shaking at 600 r/min. In the morning, plates were removed from 4°C and incubated at room temperature for 1 h while shaking at 600 r/min. After incubation, plated were removed from the shaker and the wash steps were repeated from above; 25 μL of the antibody mix was added into each well, covered (protect from light), and incubated at room temperature for 2 h shaking at 600 r/min. After incubation, plates were removed from the shaker and wash steps were repeated from above; 150 μL of read buffer was loaded into each well and read on the MSD Quickplex SQ 120 machine.

#### Angiogenesis Panel 1

Angiogenesis plates were brought to room temperature for approximately 30 min and then loaded with 150 μL of diluent, covered (protect from light), and incubated at room temperature for 1 h while shaking at 600 r/min. After incubation, plates were removed from the shaker and washed three times with 300 μL of wash buffer. Plates were turned upside down and tapped against paper towels to ensure the removal of all wash buffer from the wells; 50 μL of calibrator was added to the assigned wells in duplicate with 25 μL of undiluted sample and 25 μL of diluent. Plates were covered (protect from light) and incubated at 4°C overnight while shaking at 600 r/min. In the morning, plates were removed from 4°C and incubated at room temperature for one hour while shaking at 600 r/min. After incubation, plates were removed from the shaker and wash steps were repeated from above; 25 μL of the antibody mix was added into each well, covered (protect from light), and incubated at room temperature for 2 h shaking at 600 r/min. After incubation, plates were removed from the shaker and wash steps were repeated from above; 150 μL of read buffer was loaded into each well and read on the MSD Quickplex SQ 120 machine.

Samples were run in duplicate and three pooled control samples were run on each plate to measure inter- and intra-plate variability. MSD quantification was performed on a table stabilizer in order to reduce error in MSD plate readings.

### Plasma Sample Analysis

Protein markers measured through MSD assays were subjected to intra- and inter-plate variability tests. Intra-plate variability was assessed through two distinct methods. The first method calculated the percentage of samples for each marker that had a coefficient of variation, as determined by the duplicate runs for each sample, greater than or equal to 0.25. Markers that contained 20% of samples above this threshold were removed from further analysis. The second method ran three pooled control sample twice on the same plate (two samples each run in duplicate) to ensure consistency in final quantifications. The coefficient of variation for each of the three controls measured for each marker was then averaged together. Markers with an average coefficient of variation greater than 0.25 were excluded from the analysis. Markers that passed both criteria were included in the final analysis. Inter-plate variability was accounted for using the three pooled control samples run on each plate. Each plate control value was divided by the control mean and all three of these values for each marker were averaged together to provide a plate-scaling factor. Each value was then divided by this factor to adjust for inter-plate variability. The resulting measures were log-transformed to scale each marker to a common order of magnitude, which is required in clustering algorithms to provide equal weighting of markers. Grubb’s test was lastly applied to the data to remove outliers (Grubbs, 1950). Individual samples containing one or more outliers in the measured markers were excluded from further analysis (*n* = 7) due to their effects on clustering techniques. The final dataset consisted of 66 patient plasma samples, which were quantified for 11 plasma markers (FGF, FLT, PLGF, Tie-2, VEGF, VEGFD, MMP1, MMP3, MMP9, IL8, TNFα).

### Hierarchical Clustering Analysis

All HCAs were performed using the Matlab Statistics and Machine Learning Toolbox functions *pdist*, *linkage*, and *cluster.* Previously described HCA models were comprised of three different algorithms, distance, linkage, and clustering (Wallin et al., 2010; Damian et al., 2013; Nettiksimmons et al., 2014; Racine et al., 2016). The conventional HCA model consists of a Euclidean distance algorithm, which calculates the distance between two samples using the Euclidean distance formula (a special case of the generalized Minkowski distance formula), where the distance between observations *s* and *t* in a sample with *n* markers equals *d*_*st*_:

$$d_{s\,t}=\sqrt{\sum_{j=1}^{n}{\left|X_{s\,j}-X_{t\,j}\right|}^{2}}$$

The linkage algorithm used was Ward’s Linkage, which calculates the incremental increase in within-cluster sum of squares and links samples one at a time until all samples are combined into a single cluster (Xu and Wunsch, 2010). This method combines similar samples until all samples fall within one cluster (i.e., agglomerative hierarchical clustering). The final algorithm in the conventional HCA model used a standard agglomerative clustering approach (Racine et al., 2016).

The novel proposed HCA model uses consensus clustering as presented by Fred and Jain (2002) to combine HCA models with different distance and clustering algorithms. The distance algorithms used the Minkowski distance formula with *p* ranging from 0.1 to 2.0 in increments of 0.1. The distance between observations *s* and *t* in a sample with *n* markers equals *d*_*st*_:

$$d_{s\,t}=\sqrt[{p}]{\sum_{j=1}^{n}{\left|X_{s\,j}-X_{t\,j}\right|}^{p}}$$

Each distance algorithm’s data were then used with the weighted average linkage algorithm, which combines samples into clusters that have the smallest distance between them and determines that distance using a recursive function which treats the subset of linkages equally (Xu and Wunsch, 2010).

Lastly, data from each linkage algorithm were clustered using an inconsistency clustering algorithm. This algorithm calculates an inconsistency coefficient of a new linkage using the mean and standard deviation of the linkage heights for a specified depth (*dep*) of sub linkages within each new linkage. Clusters were formed when the inconsistency coefficient for each linkage and all sub linkages were less than a specified cutoff (*cut*) value. Each linkage algorithm output was run through multiple iterations of the inconsistency clustering algorithm with values for depth (*dep*) from 2 to 6 in increments of 1, whereas cutoff (*cut*) values were adjusted from 1.0 to 3.0 in increments of 0.1. All iterations of depth and cutoff were evaluated, and if only one cluster was formed, then that iteration was not used in the consensus clustering model. Once each clustering model was established, distances between samples were calculated based on the percentage of models in which two samples shared a cluster. Samples that shared no clusters were given a distance of 1 and, samples that were paired in the same cluster in each model were given a distance of 0. Plots of each clustering model were created using the dimensionality reduction function, t-distributed stochastic neighbor embedding (t-sne), with a random number generation seed of 10 to maintain reproducibility (Maaten, 2008). Clinical data were excluded from the clustering algorithm to avoid clusters based on clinical findings as this study sought to identify clusters of participants based on a differential level of fluid biomarkers.

### Simulated Data Generation and Analysis

Simulated data generation was performed using the Matlab Statistics and Machine Learning Toolbox function *mvnrnd*. Each simulated data experiment was run with 35 trials and each trial was initiated with a unique random number generation seed to maintain reproducibility. Generated data contained 11 variables and 100 samples per group, obtained from known distributions with the mean and sigma of each distribution differing depending on the experiment. Supplementary Tables S1–S3 detail the mean and sigma for each group within each experiment. The ARI was used to evaluate the accuracy of each clustering model by comparing each clustering result to the known cluster assignment. The ARI has a maximum value of 1 indicating that the clustering result matches perfectly to the known cluster assignment. An ARI of 0 indicates that the clustering model assigns observations to the correct cluster assignment with an equal probability as random chance. An ARI below 0 demonstrates that the clustering model is less effective than random chance at assigning observations to the correct cluster assignment (McComb, 2015).

### Statistical Analysis

Statistical analysis was performed using the Matlab Statistics and Machine Learning Toolbox and SPSS. A two-sample *t*-test using the Matlab function *ttest2* was conducted to compare the ARI means of the two HCA models for all simulated data experiments. SPSS was used for the remaining statistical tests to determine differences between clusters for each log-transformed protein marker. Levene’s test for equality of variances was performed before each two-sample *t*-test, and Satterthwaite’s *t*-test was used for any marker found to have significantly different variances. Levene’s test for homogeneity of variances based on the mean was also conducted before performing an ANOVA test for each marker and a Welch’s test for equality of means was performed for markers with non-homogeneous variances. *Post hoc* analysis was then conducted on markers which had a significant *p*-value for an ANOVA or Welch’s test. Tukey’s HSD was used for significant ANOVA tests and Dunnett T3 was used for significant Welch’s test.

## Results

### Study Population Description

Demographic and neurocognitive evaluations were obtained in 65/66 participants within our MCI-CVD cohort (Table 1). The mean age of the participants was 75.07 ± 8.14 with a female population of 47%. MMSE scores ranged from 18 to 30 with a mean of 26.86 ± 2.95, while MoCA scores ranged from 11 to 28 with a mean of 22.11 ± 3.74. Vascular risk factors including systolic blood pressure, hemoglobin A1C, and LDL cholesterol were also evaluated in our cohort (Table 1). Mean systolic blood pressure was found to be 141.33 ± 15.31 mmHg, hemoglobin A1c 6.18 ± 1.31%, and LDL cholesterol 97.44 ± 42.63 mg/dL.

**TABLE 1:** Means ± standard deviation for age, MMSE, and MoCA for the MCI-CVD cohort population in addition to percent of female participants.

	**Mean ± SDev**	**Range**
Age (years)	75.078.14	(56.99–89.22)
MMSE	26.862.95	(18–30)
MoCA	22.113.74	(11–28)
Systolic blood pressure (mmHg)	141.3315.31	(102–185)
Hemoglobin A1c (%)	6.181.31	(4.3–11.8)
LDL cholesterol (mg/dL)	97.4442.63	(22–299)
Sex	47% Female	

**TABLE 2:** Means ± SEM for clusters 1 and 2 produced by the combined HCA model.

**Biomarker**	***p*-value**	**Cluster 1 (pg/mL)**	**Cluster 2 (pg/mL)**
FGF	< 0.001	24.562.39	12.271.22
FLT	0.568	12.921.35	11.870.59
PIGF	0.093	3.520.38	2.940.11
Tie-2	0.013	500.5531.69	411.0914.77
VEGF	< 0.001	98.3917.46	43.553.63
VEGFD	0.350	560.7352.17	515.5924.35
MMP1	< 0.001	5244.74700.11	2292.86219.55
MMP3	0.005	17275.463267.49	10547.71856.39
MMP9	< 0.001	43561.188177.19	19232.981851.63
IL8	< 0.001	4.200.49	2.250.11
TNFa	0.283	1.810.15	1.630.10

*Statistical significance between groups was determined using the log transform of the data shown in Figure 4 in an independent samples *t*-test (*p* < 0.05).*

**TABLE 3:** Means ± SEM for clusters 1–4 produced by the Euclidean distance model.

**Biomarker**	***p*-value**	**Cluster 1 (pg/mL)**	**Cluster 2 (pg/mL)**	**Cluster 3 (pg/mL)**	**Cluster 4 (pg/mL)**
FGF	< 0.001	24.902.78	5.160.60	12.701.37	20.111.97
FLT	0.340	12.771.56	13.080.82	11.051.44	11.420.82
PIGF	0.342	3.530.45	2.940.17	2.790.28	3.100.16
Tie-2	0.040	512.4235.98	437.1325.44	384.2926.72	405.6421.72
VEGF	< 0.001	106.9719.29	49.976.65	27.694.39	48.565.14
VEGFD	0.258	563.8860.58	579.1846.39	469.2536.56	487.2532.33
MMP1	< 0.001	5605.20769.82	3061.39356.41	736.2771.84	2692.85285.62
MMP3	0.096	17517.093822.29	10666.691443.84	9646.521192.19	11587.351561.28
MMP9	< 0.001	46055.299378.94	25868.673771.38	17474.832787.13	14764.341748.47
IL8	< 0.001	4.490.52	2.320.18	1.780.12	2.530.18
TNFα	0.362	1.860.16	1.520.16	1.510.10	1.830.21

*Statistical significance between groups was determined using the log transform of the data shown in Figure 5 in an ANOVA followed by Tukey’s HSD for *post hoc* analysis (*p* < 0.05). Results of the ANOVA omnibus are shown in *p*-value.*

### Simulated Data Analysis

**FIGURE 1:**
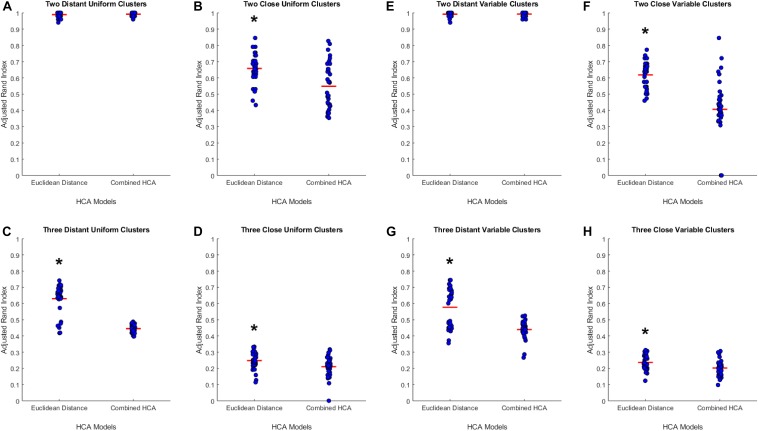
Compares the combined HCA model to the Euclidean distance model in eight different datasets. Each dataset was produced using the *mvnrnd*. The accuracy of each model was assessed using the adjusted rand index (ARI). Datasets used in **A**–**D** have identical means for each variable in each cluster. Datasets used in **E**–**H** have the same means for the first six variables and means of opposite signs for the other five variables. Means and standard deviations for each dataset are shown in Supplementary Table S1. Red horizontal lines indicate means for each model. Stars (*) indicate statistical significance between groups using an independent samples *t*-test (*p* < 0.05).

To test the applicability of the novel combined HCA model, we tested its accuracy in eight unique simulated datasets (detailed in Supplementary Table S1). We tested the novel model against an established HCA model using the ARI to measure the accuracy of each model (Figure 1). In our first experiment, we studied the accuracy of both models in a dataset with two distant uniform clusters (Figure 1A). The established HCA model using Euclidean distance showed no difference in ARI compared to the novel combined HCA model (Euclidean: 0.9892 ± 0.0029, Novel: 0.9920 ± 0.0019, *p* = 0.413). A similar result was found in a dataset with two distant variable clusters (Euclidean: 0.9920 ± 0.0023, Novel: 0.9926 ± 0.0020, *p* = 0.857) (Figure 1E). These results demonstrate that both models were able to assign each distribution to its own cluster. Next, we tested both models on a dataset with three distant uniform clusters (Figure 1C) and three distant variable clusters (Figure 1G). The established HCA model had a significantly increased ARI over the novel HCA model in both of these experiments (Euclidean: 0.6186 ± 0.0139, Novel: 0.4067 ± 0.0320, *p* < 0.001, Figure 1C) (Euclidean: 0.5767 ± 0.0200, Novel: 0.4393 ± 0.0092, *p* < 0.001, Figure 1G). These results show that the established HCA model has a higher accuracy when separating three distant clusters of normally distributed data. We then tested if the models performed differently on distributions that had more overlapping characteristics. The first experiments of these distributions were with two close uniform clusters (Figure 1B) and two close variable clusters (Figure 1F). In both experiments, the established HCA model had a higher accuracy compared to the novel HCA model (Euclidean: 0.6579 ± 0.0150, Novel: 0.5482 ± 0.0237, *p* < 0.001, Figure 1B) (Euclidean: 0.6186 ± 0.0139, Novel: 0.4067, *p* < 0.001, Figure 1F). This difference continued in the final set of experiments which used three close uniform clusters (Euclidean: 0.2477 ± 0.0087, Novel: 0.2103 ± 0.0103, *p* < 0.007, Figure 1D) and three distant variable clusters (Euclidean: 0.2370 ± 0.0080, Novel: 0.2023 ± 0.0078, *p* < 0.003, Figure 1H). These experiments show that as the distributions progressively overlap the accuracy for both models decrease and the difference between the accuracy of the models decreases as well.

### Predicted Distribution Analysis

**FIGURE 2:**
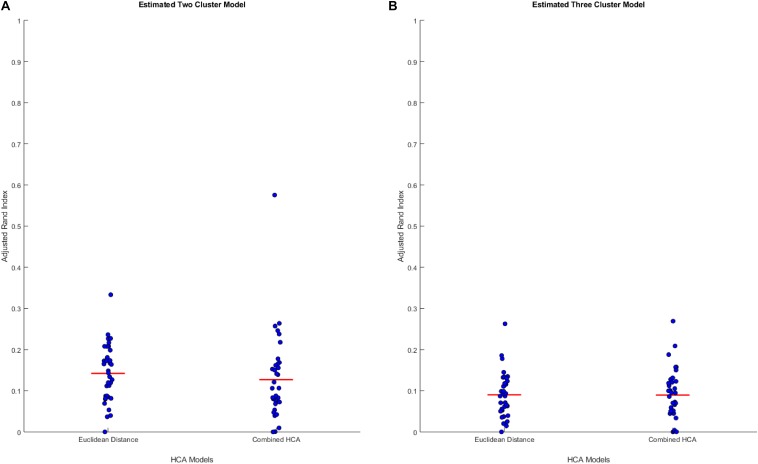
Compares the combined HCA model to the Euclidean distance model in two predicted distributions. Each dataset was produced using *mvnrnd*. The accuracy of each model was assessed using the adjusted rand index (ARI). Means and covariance matrix for each dataset are shown in Supplementary Tables S2, S3. Red horizontal lines indicate means for each model. Stars (*) indicate statistical significance between groups using an independent samples *t*-test (*p* < 0.05). **(A)** Shows estimated two cluster model, and **(B)** shows estimated three cluster model.

We hypothesized that clusters, if any, in our empirical dataset would overlap more and thus be more difficult to differentiate than those used in the previous experiments. To test the accuracy of each model in this distribution, we generated simulated data from predicted distributions based on analysis from our collaborators (detailed in Supplementary Tables S2, S3). The first experiment was based on a two-cluster model within our sample population (Figure 2A). This experiment showed no differences between the established Euclidean HCA model and the novel HCA model (Euclidean: 0.1422 ± 0.0118, Novel: 0.1270 ± 0.0181, *p* = 0.486, Figure 2A). In addition, we tested a three cluster model for our sample population and found similar results with no differences between the two models in this study (Euclidean: 0.0902 ± 0.0092, Novel: 0.0895 ± 0.0105, *p* = 0.962, Figure 2B). The data from these two experiments demonstrate the interchangeability of these two models when studying datasets with extensive overlapping distributions.

### Application of Models to Dataset

**FIGURE 3:**
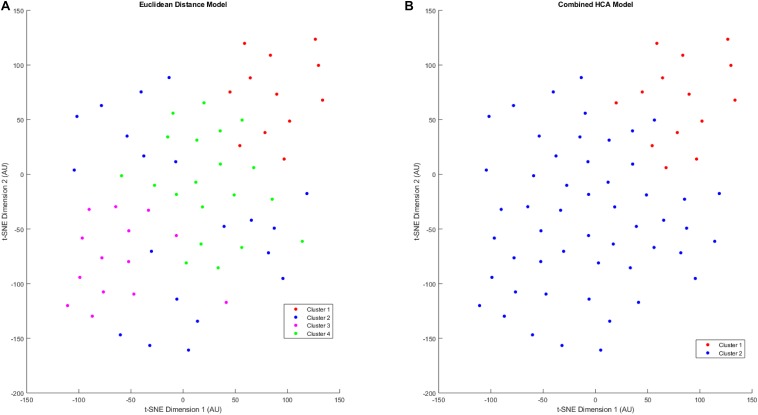
T-SNE dimensionality reduction plot of clustered data. Data were clustered using the Euclidean distance model **(A)** and the Combined HCA model **(B)**. The dataset was produced by measuring 11 inflammatory (MMP1, MMP3, MMP9, IL8, TNFα) and angiogenic proteins of interest (FGF, FLT, PIGF, Tie-2, VEGF, VEGF-D) from 66 participant plasma samples.

After validating the novel combined HCA model using predicted distributions, we applied both HCA models to our 66 patient sample (Figure 3). When the Euclidean distance HCA model was applied to our dataset, four clusters emerged (Figure 3A). Clusters 1, 3, and 4 appear to be more compact in the 2-D t-SNE dimensionality reduction plot, while cluster 2 exists along the periphery of the plot in a more scattered manner. We continued this experiment and applied the novel combined HCA model to the same dataset and uncovered two clusters (Figure 3B). Cluster 1 contains 14 samples of which 12 also appear in cluster 1 of the Euclidean distance HCA model. The other 52 samples appear in cluster 2, which is comprised of clusters 2–4 from the Euclidean distance HCA model. The similarity of these two results emphasizes the underlying distributions within this dataset.

### Characterizing Cluster Differences

**FIGURE 4:**
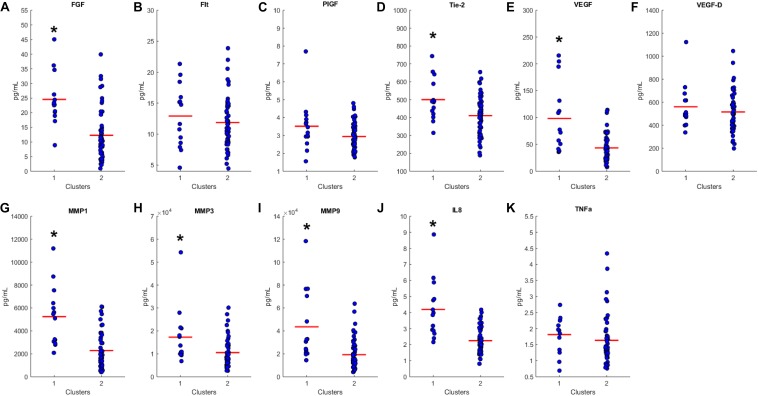
Compares clusters 1 and 2 produced from the Combined HCA model in each inflammatory and angiogenic protein measured. **(A–K)** Show mean and scatter of FGF **(A)**, FLt **(B)**, PlGF **(C)**, Tie-2 **(D)**, VEGF **(E)**, VEGF-D **(F)**, MMP1 **(G)**, MMP3 **(H)**, MM9 **(I)**, IL8 **(J)**, and TNFa **(K)**. Red horizontal lines indicate the means for each cluster. Stars (*) indicate statistical significance between groups as calculated by the log transform of the data shown using an independent samples *t*-test (*p* < 0.05). Means ± SEM are shown in Table 2.

**FIGURE 5:**
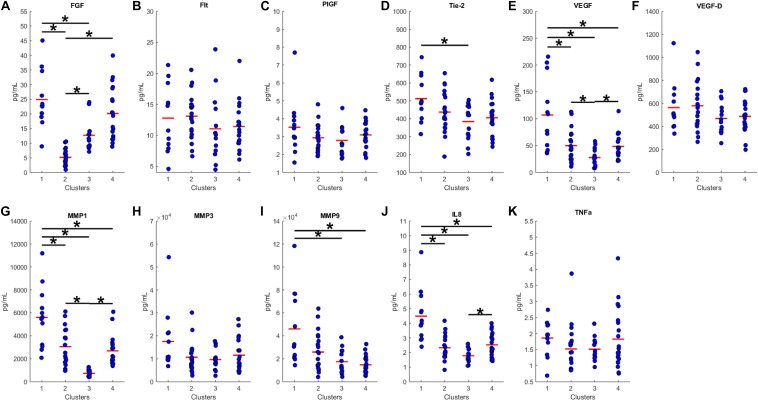
Compares clusters 1–4 produced from the Euclidean distance model in each inflammatory and angiogenic protein measured. **(A–K)** Show mean and scatter of FGF **(A)**, FLt **(B)**, PlGF **(C)**, Tie-2 **(D)**, VEGF **(E)**, VEGF-D **(F)**, MMP1 **(G)**, MMP3 **(H)**, MM9 **(I)**, IL8 **(J)**, and TNFa **(K)**. Red horizontal lines indicate the means for each cluster. Stars (*) indicate statistical significance between groups as calculated by the log transform of the data shown using ANOVA followed by Tukey’s HSD for *post hoc* analysis (*p* < 0.05). Means ± SEM are shown in Table 3.

**FIGURE 6:**
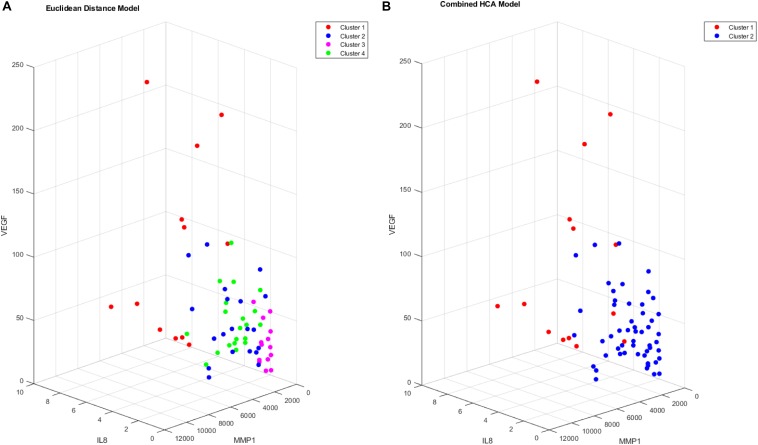
Cluster comparison of VEGF (pg/mL), MMP1 (pg/mL), and IL8 (pg/mL) for data clustered using the Euclidean distance model **(A)** and the combined HCA model **(B)** from 66 participant plasma samples.

We proceeded to analyze the differences that drive cluster differentiation. First, we examined the clusters produced by the novel HCA model (Figure 4), and found that cluster 1 was increased compared to cluster 2 in FGF (*p* < 0.001), Tie-2 (*p* = 0.013), VEGF (*p* < 0.001), MMP1 (*p* < 0.001), MMP3 (*p* = 0.005), MMP9 (*p* < 0.001), and IL8 (*p* < 0.001) (Table 3). When the clusters produced from the Euclidean distance HCA model were analyzed (Figure 5), we found a similar pattern of clusters. In this model, cluster 1 was increased compared to clusters 2–4 in VEGF (*p* = 0.006, *p* < 0.001, *p* = 0.013), MMP1 (*p* = 0.003, *p* < 0.001, *p* = 0.001), and IL8 (*p* < 0.001, *p* < 0.001, *p* < 0.001), respectively. Cluster 1 was also increased in FGF (*p* = 0.004), Tie-2 (*p* = 0.033), and MMP9 (*p* = 0.003) compared to cluster 3. The elevated level of these proteins in cluster 1 agrees with the characteristics of cluster 1 established previously with both models demonstrating a subset with significant increases in VEGF, MMP1, and IL8 compared to the other subsets (Figure 6). However, the Euclidean distance HCA model does show differences between clusters 2 and 4, which were not seen in the novel HCA model. Clusters 2 and 4 were similar in their makeup, both increased over cluster 3 in MMP1 (*p* < 0.001 and *p* < 0.001) and VEGF (*p* = 0.032 and *p* = 0.016), respectively, but different levels of FGF (*p* < 0.001). These differences lead to the possibility of four disease profiles within the MCI-CVD patient population.

## Discussion

The results of this study provide evidence supporting the use of the novel combined HCA model in datasets with extensive overlapping distributions. The results of the first set of experiments demonstrate that the Euclidean distance HCA model outperforms the novel combined HCA model in datasets with a moderate amount of overlapping distributions (Figures 1B–D,F–H). This difference is reduced as the distributions progressively increase in overlap and eventually disappears entirely in our second set of experiments involving the more complex datasets with predicted distributions (Figure 2).

It is important to note that the capacity of the two HCA models to accurately cluster data into their known distributions decreases as the datasets become more complex. In our experiments involving two distant distributions of data, both models were able to separate each distribution with an ARI approximately equal to 1 (Figures 1A,E). In experiments using our predicted distributions, the average ARI decreased to approximately 0.13 and 0.09 for the two and three cluster models, respectively (Figure 2). These findings demonstrate the limits of reliability in both HCA models and provide a measure to compare additional HCA models to in future experiments. Accounting for this accuracy is crucial when interpreting HCA results because clusters produced by the HCA model may not correspond to any true unique distribution and may simply be a subset within the normal variation of a larger distribution. Therefore, it is important to compare multiple HCA models on an unknown dataset in order to elucidate which clusters are in fact unique distributions within the dataset. Overall, results from our experiments support the interchangeability of HCA models in datasets similar to those shown in Supplementary Tables S2, S3, which allows for the use of both models in assessing clustering distributions within our dataset.

Both the Euclidean distance HCA model and the novel combined HCA model resulted in similar disease profiles within our cohort of MCI-CVD patients (Figure 3). In this study, both models classified participants into a cluster that had elevated levels of VEGF, MMP1, and IL8 compared to the other clusters (Figures 4, 5). We suspect that this disease profile seen in cluster 1 may be involved in a more active VCID process resulting in increased pathology due to the increased level of angiogenic and inflammatory markers. Clusters 2–4 in the Euclidean distance HCA model may also be clinically relevant in terms of disease pathology but require future studies to understand how these profiles may contribute to progression of VCID in a population of individuals with MCI-CVD.

## Conclusion

The usage of both the novel HCA model and a Euclidean distance HCA model identified a novel subset of patients within the MCI-CVD population. This study provides insight into a potential underlying inflammatory and angiogenic profile of disease in patients with VCID. Defining subsets of patients within this population with different disease profiles continues to be a key research objective. These profiles can provide a more complete understanding of disease progression and allow physicians and researchers to identify patients undergoing different rates of pathologic change in a prospective cohort. In the future, we hope to further clarify these profiles by combining plasma and MRI imaging biomarkers that can also be used in clinical trials as key outcome measures to determine the efficacy of novel therapeutics.

## Data Availability Statement

The datasets generated for this study are available on request to the corresponding author.

## Ethics Statement

The studies involving human participants were reviewed and approved by the University of Kentucky Institutional Review Board. The patients/participants provided their written informed consent to participate in this study.

## Author Contributions

ZW designed and executed the analyses. TS performed the assessment of analytes using MSD. DF and QC consulted on study design. LG consulted on clinical readouts. PN provided samples. FS consulted on neuropsychological assessments. GJ assessed the patients and collected all samples. DW oversaw the studies and assisted in the development of hypotheses and data interpretation.

## Conflict of Interest

The authors declare that the research was conducted in the absence of any commercial or financial relationships that could be construed as a potential conflict of interest.
