# Supplementary material for: Hierarchical Clustering Analyses of Plasma Proteins in Subjects With Cardiovascular Risk Factors Identify Informative Subsets Based on Differential Levels of Angiogenic and Inflammatory Biomarkers
Source: Front Neurosci. 2020 Feb 6;14:84. doi: 10.3389/fnins.2020.00084 (PMC7016016; doi:10.3389/fnins.2020.00084)
Supplement: Supplementary file 1 [file Table_1.DOCX]

**Supplementary Table 1**

Means and standard deviations for each cluster generated in each experiment. Distributions were generated using a multivariate normal random number generator consisting of 11 variables. The means and standard deviations for each variable are listed under Means and Sigma respectively.

**Supplementary Table 2 and 3**

Means and covariance matrix for each cluster generated in the Estimated Two Cluster model and Estimated Three Cluster model. The means for each of the 11 variables within each cluster are listed above the covariance matrices used to generate the data using multivariate normal random number generators.
